# Effect of vitamin K_2_ on the anticoagulant activity of warfarin during the perioperative period of catheter ablation: Population analysis of retrospective clinical data

**DOI:** 10.1186/s40780-016-0053-8

**Published:** 2016-08-04

**Authors:** Zhi Zhou, Ikuko Yano, Sumiko Odaka, Yosuke Morita, Satoshi Shizuta, Mamoru Hayano, Takeshi Kimura, Akinori Akaike, Ken-ichi Inui, Kazuo Matsubara

**Affiliations:** 1 Department of Clinical Pharmacology and Therapeutics, Kyoto University Hospital, Sakyo-ku, Japan; 2 Department of Pharmacology, Graduate School of Pharmaceutical Sciences, Kyoto University, Sakyo-ku, Japan; 3 Department of Clinical Pharmacy and Education, Graduate School of Pharmaceutical Sciences, Kyoto University, Sakyo-ku, Kyoto Japan; 4 Department of Cardiovascular Medicine, Graduate School of Medicine, Kyoto University, Sakyo-ku, Japan

**Keywords:** Warfarin, Vitamin K_2_, Pharmacodynamics, INR, NONMEM, Catheter ablation

## Abstract

**Background:**

Catheter ablation is a non-medication therapy for atrial fibrillation, and during the procedure, warfarin is withdrawn in the preoperative period to prevent the risk of bleeding. In case of emergency, vitamin K_2_ can be intravenously administered to antagonize the anticoagulant activity of warfarin. The aims of this study were to conduct population pharmacokinetic/pharmacodynamic modeling for retrospective clinical data and to investigate the effect of vitamin K_2_ on the anticoagulant activity of warfarin in the perioperative period of catheter ablation.

**Methods:**

A total of 579 international normalized ratio (*INR*) values of prothrombin time from 100 patients were analyzed using the nonlinear mixed-effects modeling program NONMEM. A 1-compartment model was adapted to the pharmacokinetics of warfarin and vitamin K_2_, and the indirect response model was used to investigate the relationship between plasma concentration and the pharmacodynamic response of warfarin and vitamin K_2_. Since no plasma concentration data for warfarin and vitamin K_2_ were available, 3 literally available pharmacokinetic parameters were used to simultaneously estimate 1 pharmacokinetic parameter and 5 pharmacodynamic parameters.

**Results:**

The population parameters obtained not only successfully explained the observed *INR* values, but also indicated an increase in sensitivity to warfarin in patients with reduced renal function. Simulations using these parameters indicated that vitamin K_2_ administration of more than 20 mg caused a slight dose-dependent decrease in *INR* on the day of catheter ablation and a delayed *INR* elevation after warfarin re-initiation.

**Conclusions:**

A pharmacokinetic/pharmacodynamic model was successfully built to explain the retrospective *INR* data during catheter ablation. Simulation studies suggest that vitamin K_2_ should be administered with care and that more than 20 mg is unnecessary in the preoperative period of catheter ablation.

## Background

Atrial fibrillation is the most common sustained cardiac arrhythmia and a major cause of stroke [1, 2]. In order to prevent stroke, an anticoagulant drug, warfarin, is usually used since aspirin was proven ineffective in retrospective analyses [3]. The anticoagulant effect of warfarin does not always correlate with its dose, and polymorphisms in cytochrome P450 (CYP) 2C9 and vitamin K epoxide reductase complex subunit 1 (VKORC1) genes have been proven to influence interindividual variability in the optimal doses, in addition to patients’ primary diseases and characteristics such as age or ethnicity [4, 5]. In Japanese patients, warfarin dose adjustments based on their prothrombin time, an international normalized ratio (*INR*) of 1.6–2.6 (age ≥ 70 years) or 2.0-3.0 (age < 70 years), are recommended for effective therapy to avoid life-threatening bleeding [6, 7]. When hemorrhagic complications occur, warfarin withdrawal is required and vitamin K_2_ or fresh frozen plasma administration is recommended [8–10].

In atrial fibrillation treatment, antiarrythmic agents are often used, while catheter ablation is also an available option as a non-medication therapy [2]. When catheter ablation, an invasive procedure for complete cure of atrial fibrillation, is selected, anticoagulant therapy with warfarin is withdrawn in the preoperative period to prevent the risk of bleeding, although catheter ablation is sometimes performed in periprocedural therapeutic anticoagulation with warfarin if possible. In some patients, discontinuation of warfarin is not sufficient to lower the *INR* to the required level before catheter ablation. In such cases, vitamin K_2_ is intravenously administered to antagonize the anticoagulant activity of warfarin resulting in prompt recovery of *INR* to a safe level. Some reports have mentioned the use of pharmacokinetic/pharmacodynamic models for an anticoagulant drug and have conducted population analyses; however, only warfarin was investigated using these models [11, 12]. The effect of vitamin K_2_ dose on controlling the anticoagulant activity of warfarin during the perioperative period of catheter ablation has not yet been reported. The aims of this study are to build a population pharmacokinetic/pharmacodynamic model not only for warfarin, but also for vitamin K_2_, by using routine clinical data of patients who had been diagnosed with atrial fibrillation and received a catheter ablation, and to obtain information on the optimal vitamin K_2_ dose in the preoperative period before catheter ablation.

## Methods

### Patients and data studied

We retrospectively collected data from patients who have had a catheter ablation for atrial fibrillation at the Department of Cardiovascular Medicine, Kyoto University Hospital from January to December in 2008. During this period, 126 Japanese patients underwent catheter ablation, and 111 of these patients were treated with warfarin on the day of admission. A total of 100 patients whose *INR* values were between 1.0 and 3.0 in the hospitalization period were included in this study. We used 579 *INR* values obtained from 100 patients during the perioperative period. Clinical laboratory data and medication history for the patients studied were collected from electrical medical records. No patients were taking any medications that may have clinically significantly altered the pharmacokinetics of warfarin, except 4 patients with amiodaron and 1 patient with bucolome [13, 14].

### Pharmacokinetic/pharmacodynamic model building

**Fig. 1:**
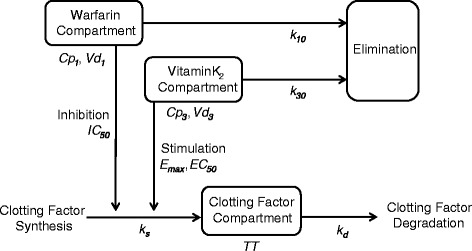
Pharmacokinetic/pharmacodynamic model of warfarin and vitamin K_2_. In this model, *Cp*_*1*_ and *Cp*_*3*_ represent the plasma concentration of warfarin and vitamin K_2_, respectively; *Vd*_*1*_ and *Vd*_*3*_ represent the distribution volume; and *k*_*10*_ and *k*_*30*_ represent the first-order elimination rate constant for each drug. The zero-order synthesis and first-order degradation rate constant for clotting factors are shown as *k*_*s*_ and *k*_*d*_, respectively, and *IC*_*5*0_, *E*_*max*_ and *EC*_*50*_ represent 50 % inhibitory concentration of warfarin, maximum effect of vitamin K_2_ and 50 % effective concentration of vitamin K_2_, respectively

A 1-compartment model was adopted to the pharmacokinetics of warfarin and vitamin K_2_ as follows (Fig. 1):

1 $$ d\left(C{p}_1*V{d}_1\right)/dt = -{k}_{10}*\left(C{p}_1*V{d}_1\right) $$

2 $$ d\left(C{p}_3*V{d}_3\right)/dt = -{k}_{30}*\left(C{p}_3*V{d}_3\right) $$

where *Cp*_*1*_ and *Cp*_*3*_ represent the plasma concentration of warfarin and vitamin K_2_, respectively; and *Vd*_*1*_ and *Vd*_*3*_ represent the distribution volume; and *k*_*10*_ and *k*_*30*_ represent the elimination rate constant for each drug, respectively. Since no plasma concentration data were available for warfarin and vitamin K_2_, and *INR* values were the available data for this study, reported pharmacokinetic parameters for warfarin in Japanese patients [11] and the distribution volume for vitamin K_2_ in the product information (Eisai Co., Ltd., Tokyo, Japan) were used in the analysis: *k*_*10*_ = 0.0129 (1/h), *Vd*_*1*_ = 0.183 (L/kg) and *Vd*_*3*_ = 0.051 (L/kg). Therefore, *k*_*30*_ was the only pharmacokinetic parameter to be estimated in this analysis.

The indirect response model was used to explain the relationship between plasma concentration and pharmacodynamic response of warfarin and vitamin K_2_ [11, 14–16]. In this model, the amount of clotting factors was described using a zero-order synthesis rate constant (*k*_*s*_) and a first-order degradation rate constant (*k*_*d*_) under the hypothesis that coagulant activity was proportional to the amount of clotting factors (Fig. 1). Since both warfarin and vitamin K_2_ target the same enzyme that is responsible for clotting factor synthesis [17], the maximum effect models were adopted to describe stimulatory and inhibitory activities of these drugs, respectively, as follows:

3 $$ d(TT)/dt={k}_s\ast \left(1{\textstyle \hbox{-} }C{p}_1/\left(C{p}_1+I{C_5}_0\right)+{E}_{max}\ast C{p}_3/\left(C{p}_3+E{C}_{50}\right)\right){\textstyle -}{k}_d\ast TT $$

where *k*_*s*_, *k*_*d*_, *IC*_*50*_, *E*_*ma*x,_ and *EC*_*50*_ represent synthesis rate constant (%/h), degradation rate constant (1/h), 50 % inhibitory concentration of warfarin (μg/mL), maximum effect of vitamin K_2_ (no unit), and 50 % effective concentration of vitamin K_2_ (μg/mL) were used, respectively (Fig. 1). The Hill coefficient used in the previous study [11] was not included in the present model to simplify the pharmacodynamic model. Since only *INR* values were collected in this study, thrombotest (*TT*) values were calculated according to Equation 4 using values provided from literature [18]:

4 $$ TT\left(\%\right)=23.77\ast INR/\left(INR{\textstyle -}0.8085\right){\textstyle -}0.09807\ast INR{\textstyle -}23.04 $$

Since the predicted values were outputted by the nonlinear mixed-effects modeling program (NONMEM) [19] using *TT* values, these were then converted into *INR* values when necessary by solving the quadratic equation obtained from Equation 4.

The population pharmacokinetic and pharmacodynamic analysis was performed using the NONMEM (version VI), using the first-order conditional estimation method [19]. In this study, exponential error models for both inter- and intraindividual variability were chosen as follows:

5 $$ {P}_{ij}={P}_{pop,i}\ast exp\left({\eta}_{ij}\right) $$

6 $$ T{T_j}_k=T{T^{\ast}}_{jk}\ast exp\left({\epsilon}_{jk}\right) $$

where *P*_*ij*_ is the *i*-th individual pharmacokinetic or pharmacodynamic parameter for patient *j*; *P*_*pop*,*i*_is the *i*-th population mean parameter; and η_*ij*_ is the individual random perturbation from the population mean parameter that is distributed with a mean of zero and variance ω_ι_^*2*^. *TT*_*jk*_ is the observed *TT* value at time *k* for patient *j*; TT^*^_jk_ is the corresponding predicted *TT* value; and ε_*jk*_ represents the independent identically distributed error with a mean of zero and variance of σ^*2*^ for the *TT* value.

The number of η used in the model was determined by the method of minimum Akaike information criterion (*AIC*) estimation [20].

7 $$ AIC=OBJ + 2M $$

where *OBJ* is the objective function values calculated using the NONMEM and *M* is the number of independently adjusted parameters within the model.

Next, the influence of renal function on each parameter was examined using Equation 8, by the forward selection method.

8 $$ {P}_{pop,i}={P^{*}}_{pop,i}*{\theta}^{RF} $$

where *RF* = 1 if serum creatinine was higher than our in-hospital reference value, namely 1.1 mg/dL or higher for men, and 0.8 mg/dL or higher for women, otherwise *RF* = 0. P^*^_pop,*i*_ is the *i*-th population mean parameter in the patient whose serum creatinine is within our in-hospital reference value. The parameter set that had the smallest objective function value was selected, and the null hypothesis that θ was not statistically different from unity was examined using the likelihood ratio test. A difference of 7.88 in *OBJ* with 1 degree of freedom was used to measure statistical significance (*P* < 0.005 by the chi-squared distribution).

### Simulation for *INR* transition

#### (A) Effect of vitamin K_2_ dose

Simulations were carried out using the obtained population mean parameters based on a typical patient whose body weight was 50 kg with/without renal failure. The maintenance dose of warfarin was set to 3 mg/day (7 PM) and was stopped on day −1 (the day prior to the operation), and 5 mg/day was administered for 2 days after the operation as a loading dose, followed by a maintenance dose of 3 mg/day. Vitamin K_2_ was administered at 20 mg 0, 1, 2, or 3-times every 4 hours after 4 PM on day −1 with the total dose administered ranging from 0 mg to 60 mg.

For quantitative evaluation, we obtained 4 parameters, namely ∆*INR*, *1st loading*, *95* % *recovery*, and *INR*/*day*. The ∆*INR* represents the difference in *INR* values between before warfarin withdrawal and before the loading dose; the *1st loading* represents an *INR* increase after the first warfarin loading dose; and the *95* % *recovery* represents the time needed for *INR* elevation in the postoperative period up to 95 % of the preoperative steady state *INR* value. In addition, *INR*/*day* was calculated by dividing ∆*INR* by *95* % *recovery* (day).

#### (B) Effect of warfarin dose

Simulations with various warfarin maintenance doses were conducted. As a maintenance dose, 3 to 6 mg of warfarin was administered and it was stopped on day −1 without vitamin K_2_ administration. Warfarin (2 mg) was added to each maintenance dose as a loading dose, and it was administered for 2 days after the operation, followed by each maintenance dose. Cases where 20 to 60 mg of vitamin K_2_ was administered were also simulated.

#### (C) Effect of interindividual variability

Simulations were also conducted using several parameter sets in which 1 of the mean parameters was altered using the interindividual variability (+ or – ω) from the population mean value. Warfarin and vitamin K_2_ doses were set to 3 and 20 mg, respectively, in each simulation.

## Results

### Patients’ characteristics and *INR* transitions

**Fig. 2:**
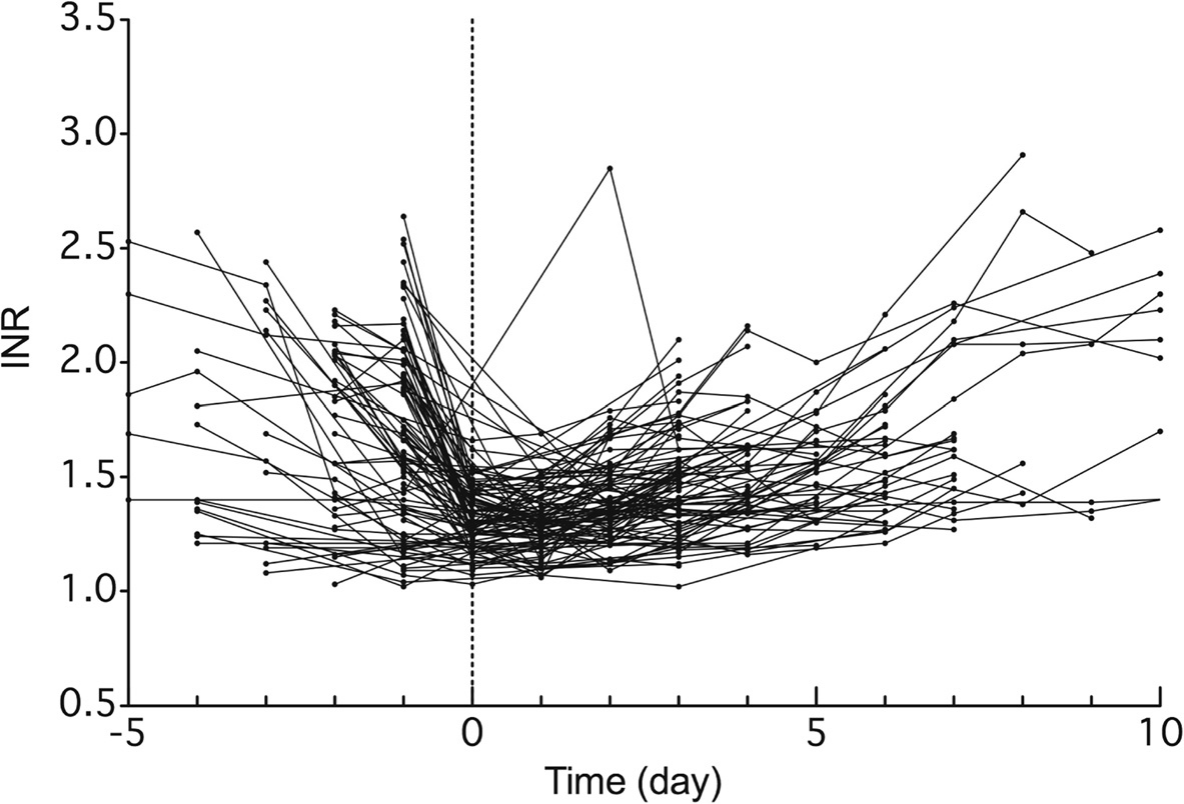
Observed *INR* values *versus* time in 100 patients who received the catheter ablation. Time 0 shows the day of catheter ablation. Data from the same patient are connected

Table 1 shows the characteristics of patients used in this study. Each patient received anticoagulant therapy of 1 to 7 mg/day of warfarin to prevent thromboembolic events. The median initial *INR* value on the day of admission was 1.76, and the median maintenance dose before hospitalization and the median loading doses of warfarin after the operation were 3 and 5 mg, respectively. To antagonize warfarin after its withdrawal in the preoperative period, a total of 20 to 70 mg of vitamin K_2_, determined by the physician responsible, was intravenously administered to 76 patients before the operation. There were 4 patients with a total bilirubin concentration greater than our in-hospital reference value, but not substantially higher. Eight patients had an albumin concentration lower than our in-hospital reference values. Twenty-two patients had a serum creatinine concentration greater than our in-hospital reference value. Twenty-six patients had an estimated glomerular filtration rate from 30 – 60 mL/min/1.73 m^2^, and only 2 patients had below 30 mL/min/1.73 m^2^. Figure 2 shows the *INR* transitions of each patient from day −5 to day 10, where the day of operation was day 0. The *INR* values decreased during the preoperative period and gradually increased again during the postoperative period.

**Table 1:** Patient characteristics

Characteristics	Number or median (min-max)
Total number of patients (M/F)	100 (70/30)
Age (years)	64 (31–80)
Body weight (kg)	63.8 (34.9-92.6)
Initial *INR*	1.76 (1.03-2.64)
Warfarin maintenance dose (mg)	3.0 (1.0-7.0)
Warfarin loading dose (mg)	5.0 (1.0-9.0)
Number of patients treated with vitamin K_2_	76
Total dosage of vitamin K_2_ (mg)	40 (20–70)
20 mg	19
30 mg	2
40 mg	35
60 mg	19
70 mg	1
Total bilirubin concentration (mg/dL)	0.7 (0.3-1.7)
Serum albumin (g/dL)	4.3 (3.6-5.0)
Serum creatinine concentration (mg/dL)	0.8 (0.5-9.6)
Estimated glomerular filtration rate (mL/min/1.73 m^2^)	67.7 (5–120)

*INR*, prothrombin internationalized ratio

### Model development

When interindividual variability was considered for all population pharmacokinetic/pharmacodynamic mean parameters (η = 6), *AIC* was 3398. To simplify the model in which only η_*ks*_ and η_*IC50*_ were included (η = 2), *AIC* was 3394, and was decreased to 3393 when another η for *k*_*30*_ was included in the model (η = 3). Thus, the model with the minimum *AIC* value was adopted, which reflected the interindividual variability of *k*_*s*_, *IC*_*50*,_ and *k*_*30*_.

Next, a search for covariates of population mean parameters was conducted using the forward selection method. When the effect of serum creatinine on each population mean parameter was examined, significant effects of renal function on *k*_*s*_, *k*_*d*_, and *IC*_*50*_ were observed (*P* < 0.005). Since the effect on *IC*_*50*_ showed the largest −2 log likelihood difference (−*2LLD*) of 27.2, this effect was incorporated into the second step. At the second step, additional effects of renal function on other parameters were examined, but no significant differences were observed (−*2LLD* < 0.61). We also examined the effect of renal function on the *IC*_*50*_ using the value of estimated glomerular filtration rate, but the model fitting was better in the model using serum creatinine. Therefore, we chose the model in which only *IC*_*50*_ was affected by renal function as follows:

9 $$ d(TT)/dt={k}_s\ast \left(1-C{p}_1/\left(C{p}_1+I{C}_{50}\ast {\theta}^{RF}\right)+{E}_{max}\ast C{p}_3/\left(C{p}_3+E{C}_{50}\right)\right){\textstyle -}{k}_d\ast TT $$

There were only 4 patients out of 100 patients whose total bilirubin concentration exceeded our in-hospital reference value, and those values were not remarkably high. Therefore, the effect of hepatic function on population mean parameters was not further examined. The anticoagulant effect of warfarin is generally considered to be associated with its unbound plasma concentration [10]. We examined the effect of serum albumin concentration on the *IC*_*50*_ or *k*_*10*_, but we could not obtain any significant effects.

Table 2 shows the final population mean parameters obtained and inter- and intraindividual variability. The interindividual variability for *k*_*s*_, *IC*_*50*_ and *k*_*30*_ were 26.5 %, 37.9 %, and 41.4 %, respectively, and intraindividual variability was 28.2 % as a coefficient of variation (CV). In patients with decreased renal function, the *IC*_*50*_ value was reduced to 61.4 % of those with normal renal function, suggesting enhanced sensitivity to warfarin.

**Table 2:** Final population pharmacokinetic and pharmacodynamic parameters

Mean parameters	Estimate	RSE
*k*_*s*_ (%/h)	3.97	17.5
*k*_*d*_ (1/h)	0.0611	9.90
*IC*_*50*_ (μg/mL)	0.604	24.5
*E*_*max*_	0.324	15.9
*EC*_*50*_ (μg/mL)	5.30	17.6
*k*_*30*_ (1/h)	0.0194	19.2
θ	0.614	13.9
Interindividual variability	Estimate (CV%)	RSE
ω_*ks*_^2^	0.0704 (26.5)	25.6
ω_*IC50*_^2^	0.144 (37.9)	43.3
ω_*k30*_^2^	0.171 (41.4)	85.3
Residual variability (%)	Estimate (CV%)	RSE
σ^2^	0.0798 (28.2)	11.8

*k*_*s*_, synthesis rate constant; *k*_*d*_, degradation rate constant; *IC*_*50*_, 50 % inhibitory concentration of warfarin; *E*_*max*_, maximum effect of vitamin K_2_; *EC*_*50*_, 50 % effective concentration of vitamin K_2_; *k*_*30*_, elimination rate constant of vitamin K_2_; θ, a factor for the effect of decreased renal function on *IC*_*50*_; RSE, relative standard error

### Validity of population mean parameters

**Fig. 3:**
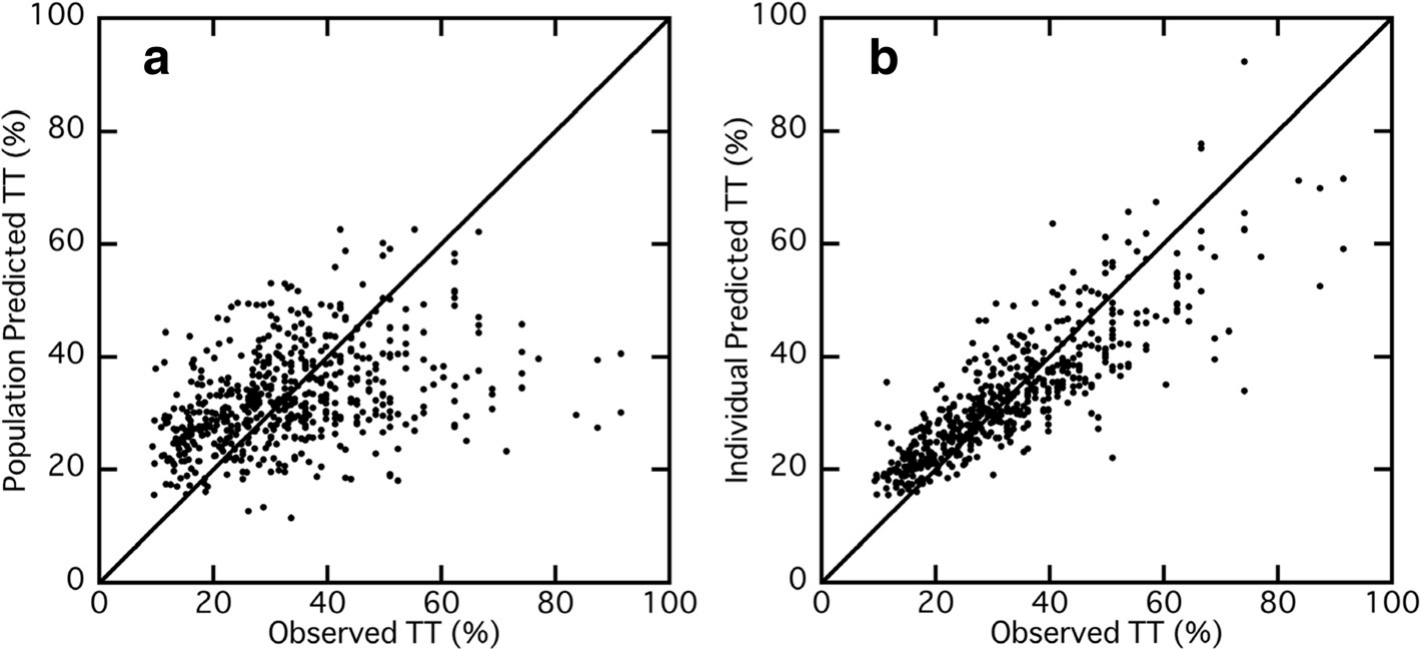
Population predicted thrombotest (*TT*, A) or individual predicted *TT* (B) versus observed *TT* in each patient. The unit line is shown

**Fig. 4:**
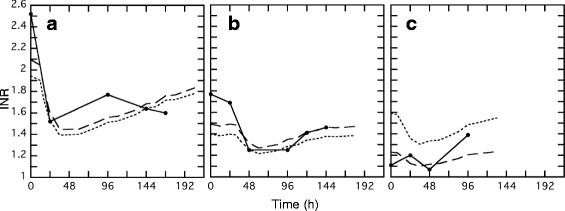
Prediction of *INR* values by the population mean parameters and by the post-hoc empirical Bayesian method in 3 typical patients. The continuous, dashed and broken lines show the observed and predicted *INR* values using population mean parameters and using the Bayesian method, respectively. Three patients were randomly selected from the database based on the *INR* values (high, median, and low) on the day of admission

Figure 3 shows the plot of population or individual (post-hoc Bayesian) predicted *versus *observed *TT*. Although there was significant variability between the population predicted and observed *TT*, each plot individually predicted by the Bayesian method was closer to the unit line. The validation of the final population parameters was further confirmed by comparing the predicted *INR* values *versus *observed *INR* values (Fig. 4). Three typical patients were randomly selected each from 3 different groups classified by *INR* values on the day of admission (high, median and low), and their predicted values were compared to the observed values. The time course profiles predicted by the Bayesian method were closer to the observed values than those predicted by the population mean parameters were, although there was still some discrepancy between these plots.

### Effect of renal function on *INR* transition

**Fig. 5:**
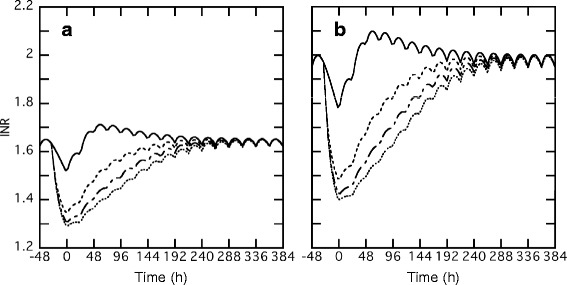
Effect of vitamin K_2_ doses on the *INR* transition in the perioperative period in a typical patient with normal **a** or decreased renal function **b**. A typical patient with body weight of 50 kg was administered warfarin and vitamin K_2_. In each simulation, the maintenance dose of warfarin was set to 3 mg/day and was stopped on day −1, and loading of 5 mg/day was carried out twice after catheter ablation, followed by the maintenance dose of 3 mg/day. Vitamin K_2_ was administered before catheter ablation up to 3 times 20 mg each as total of 0 to 60 mg. Continuous, dashed, dash-dotted and dotted lines show the administration of 0, 20, 40 and 60 mg of vitamin K_2_, respectively

Figure 5 shows the simulation curves for the effect of renal function. In a patient with decreased renal function, the *INR* value at a steady state rose from 1.65 in a patient with normal renal function to 1.99 with a maintenance dose of 3 mg/day (Fig. 5a). The *INR* transitions of a patient with decreased renal function showed more dynamic changes with variable vitamin K_2_ doses than those with normal renal function in the perioperative period. Table 3A shows the values from quantitative evaluation of Fig. 5. The ∆*INR* increased depending on the total dose of vitamin K_2_, while *95* % *recovery* was remarkably prolonged by the increased dose of vitamin K_2_. Specifically, without the administration of vitamin K_2_ to a patient with normal renal function, the *95* % *INR recovery* was 8 h, while it increased to 100 h when 20 mg of vitamin K_2_ was administered. The calculated *INR*/*day* also decreased from 0.39 (0 mg of vitamin K_2_) to 0.072 (20 mg of vitamin K_2_) in patients with normal renal function. In patients with decreased renal function, a similar but greater *INR* change compared with those with normal renal function is shown in Fig. 5 and Table 3A.

**Table 3:** Quantitative evaluation of the simulated *INR* transitions corresponding to Figs. 5 and 6

(A) Effect of renal function on *INR* transitions.									
Renal Function	Normal					Decreased renal function			
Vitamin K_2_ (mg)	0	20		40	60	0	20	40	60
Δ*INR* (×10^−1^)	1.30	3.02		3.41	3.58	2.15	5.11	5.73	5.98
*1st Loading* (×10^−1^)	0.72	0.40		0.23	0.15	1.12	0.55	0.30	0.17
*95* % *Recovery* (h)	8	100		148	172	16	126	174	200
*INR*/*day* (×10^−1^)	3.90	0.72		0.55	0.50	3.23	0.97	0.79	0.72
(B) Effects of combinations of various warfarin maintenance doses and vitamin K_2_ doses on *INR* transitions.									
Warfarin (mg)	3					4			
Vitamin K_2_ (mg)	0	20		40	60	0	20	40	60
Δ*INR* (×10^−1^)	1.30	3.02		3.41	3.58	1.75	4.10	4.62	4.83
*1st Loading* (×10^−1^)	0.72	0.40		0.23	0.15	0.77	0.38	0.20	0.11
*95* % *Recovery* (h)	8	100		148	172	26	126	172	196
*INR*/*day* (×10^−1^)	3.90	0.72		0.55	0.50	1.62	0.78	0.64	0.59
Warfarin (mg)	5					6			
Vitamin K_2_ (mg)	0	20		40	60	0	20	40	60
Δ*INR* (×10^−1^)	2.21	5.26		5.90	6.16	2.66	6.49	7.24	7.54
*1st Loading* (×10^−1^)	0.83	0.38		0.17	0.08	0.89	0.38	0.15	0.05
*95* % *Recovery* (h)	28	150		196	206	32	158	200	224
*INR*/*day* (×10^−1^)	1.89	0.84		0.72	0.72	2.00	0.99	0.87	0.81
(C) Effect of interindividual variability on *INR* transitions.									
	*ks*			*IC*_*50*_			*k*_*30*_		
	+ω	0	-ω	+ω	0	-ω	+ω	0	-ω
Δ*INR* (×10^−1^)	2.37	3.02	4.16	2.20	3.02	5.04	2.94	3.02	3.10
*1st Loading* (×10^−1^)	0.31	0.40	0.55	0.31	0.40	0.54	0.55	0.40	0.26
*95* % *Recovery* (h)	82	100	102	76	100	126	58	100	198
*INR*/*day* (×10^−1^)	0.69	0.72	0.98	0.69	0.72	0.96	1.22	0.72	0.38

*∆INR*, a decrease before and after warfarin withdrawal; *1st Loading*, an INR increase by the first warfarin loading; *95 % Recovery*, a time to elevate to the 95 % of the INR value before warfarin withdrawal; *INR/day*, ∆INR divided by 95 % Recovery

### Effect of warfarin dose on *INR* transition

**Fig. 6:**
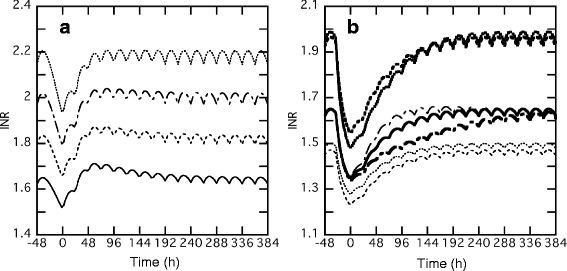
Effect of warfarin doses **a** or interindividual variability of pharmacokinetic and pharmacodynamic parameters **b** on *INR* transition in the perioperative period. In panel A, *INR* transitions were simulated wherein warfarin maintenance doses ranged from 3 to 6 mg without vitamin K_2_ administration. Warfarin was stopped on day −1, and resumed after catheter ablation with the addition of 2 mg to each maintenance dose as a loading dose twice, followed by each maintenance dose. Continuous, dashed, dash-dotted, and dotted lines show the simulations with the administration of 3, 4, 5, and 6 mg of warfarin, respectively. In panel B, *INR* transitions were simulated using parameter sets in which 1 of the parameters was changed based on interindividual variability (ω) from the population mean value. The warfarin maintenance dose and the vitamin K_2_ dose were set to 3 mg and 20 mg, respectively. The continuous, dotted, dashed, and dash-dotted lines show the simulations using all population mean values, and changed parameters for *k*_*s*_, *IC*_*50*,_ and *k*_*30*_, respectively. The fine and bold lines show the simulations using + and - ω change from each population mean value, respectively

Figure 6a shows the simulation curves with various warfarin maintenance doses. The *INR* values increased almost directly according to the increase in warfarin dose. In Table 3B, each quantitative index in Fig. 6a is shown, and the cases where 20 to 60 mg of vitamin K_2_ was administered are indicated. The ∆*INR* increased, ranging from 0.130 to 0.754 depending on both the warfarin maintenance dose and the vitamin K_2_ total dose. The *95* % *recovery* depended both on the warfarin maintenance dose and on the vitamin K_2_ total dose.

### Effect of interindividual variability on *INR* transition

Figure 6b shows the effects of interindividual variability on *INR* transition. The simulated curves suggested that the interindividual variability of *k*_*30*_ had a relatively small effect on *INR* variability, while *k*_*s*_ and *IC*_*50*_ had greater effects although they varied by 26.5 % or 37.9 %, respectively, from each population mean value. The *INR* values under a warfarin maintenance dose of 3 mg ranged from 1.47 to 1.98, and *INR* values after warfarin withdrawal ranged from 1.23 to 1.55, depending on *k*_*s*_, *IC*_*50*,_ and *k*_*30*_ values. Table 3C shows quantitative indices of the results of Fig. 6b. The ∆*INR* values ranged from 0.237 to 0.416, from 0.220 to 0.504, and from 0.294 to 0.310, when *k*_*s*_, *IC*_*50*_, and *k*_*30*_ were increased or decreased by the interindividual variability from the population mean value, respectively. The interindividual variability of *k*_*s*_, *IC*_*50*_, and *k*_*30*_ had similar effects on the *1st loading*. Unlike the ∆*INR* values, the interindividual variability of *k*_*s*_ and *IC*_*50*_ had a small effect on the *95* % *recovery*, while the *k*_*30*_ value strongly affected the *95* % *recovery*.

## Discussion

It is widely known that the warfarin dose suitable for a patient varies among individuals and that careful monitoring of its anticoagulant activity is necessary for preventing excessive anticoagulation or hemorrhagic events [6, 7]. Vitamin K_2_ can effectively antagonize warfarin, for example, in the preoperative period and when life-threatening bleeding occurs [9]. Although the recommended dose of vitamin K_2_ was under 5 mg [9], 20–70 mg of vitamin K_2_ was administered to decrease the *INR* value in the preoperative period (Table 1). Thus, caution must be exercised to find a balance between over- and under-coagulation. The pharmacokinetics and pharmacodynamics of warfarin have been studied since 1960’s [11, 12, 14, 21, 22], while combined pharmacokinetic/pharmacodynamic analyses of both warfarin and vitamin K formulations have not yet been reported. In the present study, we built a model that describes the pharmacokinetics/pharmacodynamics of these drugs for the first time. However, because this is a retrospective study wherein only patients’ pharmacodynamic data were used and because we converted the *INR* values to *TT* values while calculating the pharmacokinetic/pharmacodynamic parameters, special attention should be paid when drawing conclusions from the results obtained herein. Additionally, the obtained pharmacokinetic and pharmacodynamic parameters should be carefully treated, since these values greatly depended on the fixed pharmacokinetic parameters of warfarin and vitamin K_2_ in the model.

Final population pharmacokinetic/pharmacodynamic parameters had reasonably small relative standard errors except ω_*k30*_^2^ (Table 3), and both individual predicted *TT* and *INR* values were well correlated with the observed values (Figs. 3 and 4), indicating that reliable population mean parameters were obtained in this study. Some patients had the *INR* values between 1.0-1.5 on the day of admission (Fig. 2). We could not check drug compliance in the patients before the hospitalization, but good compliance was expected in the hospital. Since the prediction bias of the *TT* was not observed against the time (data not shown), effects of non-compliance on the present results were considered to be small. Since coadministration of amiodarone or bucolome was reported to inhibit the warfarin metabolism mediated by CYP2C9 [13, 14], we examined the effect of these drugs on the *k*_*10*_. Although the coadministration of these drugs decreased *k*_*10*_, this effect did not reach a statistical significance level (−2LLD = 7.61 < 7.88). Therefore, we did not include the effect of amiodarone and bucolome in the final model. The estimated population mean parameters for *k*_*s*_, *k*_*d*,_ and *IC*_*50*_ were similar to those in a previous report [11], and interindividual variability for *k*_*s*_, *IC*_*50*_, and *k*_*30*_ was minimal, although the intraindividual variability was quite significant.

The several simulations of *INR* transition by the obtained population pharmacokinetic/pharmacodynamic parameters showed that vitamin K_2_ could antagonize the anticoagulant activity of warfarin in a dose-dependent manner. While more than 20 mg of vitamin K_2_ showed only a small effect on the extent of *INR* decreases in the preoperative period, the time required for warfarin to exert its anticoagulation activity again in the postoperative period depended on the total dose of vitamin K_2_. An inability to anticoagulate promptly after the operation may lead to prolonged hospitalization and consequently decrease patients’ quality of life, as well as increase medical costs. Although it is important to examine the effect of less than 20 mg vitamin K_2_ on *INR*, we could not obtain clinical data using less than 20 mg vitamin K_2_. Effects of lower dose of vitamin K_2_ on *INR* remains to be examined in a future study.

In this study, we clarified the enhanced anticoagulant activity of warfarin in patients with decreased renal function. Warfarin is well known to inhibit the vitamin K-dependent synthesis pathway of coagulation factors in the liver and to be degraded in the liver [10]. Thus, great caution is required while using warfarin in patients with hepatic disorders [10]. According to the package insert of warfarin, caution is also required while use in those with renal dysfunction. Recent studies reported that renal function influences warfarin responsiveness and hemorrhagic complications [23, 24]. The maintenance warfarin dose was positively correlated with kidney function in Japanese patients [25]. Precise mechanisms for the enhanced sensitivity to warfarin in patients with decreased renal function should be investigated further in future studies.

## Conclusions

We built and analyzed a pharmacokinetic/pharmacodynamic model of both warfarin and vitamin K_2_ by using retrospective clinical data during the catheter ablation. Simulations using the obtained population pharmacokinetic/pharmacodynamic parameters indicated that vitamin K_2_ should be administered with care and that more than 20 mg is unnecessary in the preoperative period of catheter ablation. Low-dose (5 mg or less) of vitamin K is recommended in the guideline [9].

## Abbreviations

*AIC*, Akaike information criterion; Cp, plasma concentration; CYP, cytochrome P450; *EC*_*50*_, 50 % effective concentration; *E*_*max*_, maximum effect; *IC*_*50*_, 50 % inhibitory concentration; *INR*, international normalized ratio; *k*_*d*_, degradation rate constant; *k*_*s*_, synthesis rate constant; *LLD*, log likelihood difference; *OBJ*, objective function; *TT*, thrombotest; *Vd*, distribution volume, *k*, elimination rate constant; VKORC1, vitamin K epoxide reductase complex subunit 1.
